# Application of artificial intelligence in the assessment of thyroid eye disease (TED) - a scoping review

**DOI:** 10.3389/fendo.2023.1300196

**Published:** 2023-12-20

**Authors:** Chiaw-Ling Chng, Kaiping Zheng, Ann Kerwen Kwee, Ming-Han Hugo Lee, Daniel Ting, Chen Pong Wong, Guoyu Hu, Beng Chin Ooi, Si Wei Kheok

**Affiliations:** ^1^ Department of Endocrinology, Singapore General Hospital, Singapore, Singapore; ^2^ School of Computing, National University of Singapore, Singapore, Singapore; ^3^ Oculoplastic Department, Sydney Eye Hospital, Sydney, SW, Australia; ^4^ Singapore Eye Research Institute, Singapore National Eye Centre, Singapore, Singapore; ^5^ Department of Neuroradiology, Singapore General Hospital, Singapore, Singapore

**Keywords:** Graves’ ophthalmology, Graves orbitopathy, thyroid eye disease, artificial intelligence, convolutional neural networks

## Abstract

**Background:**

There is emerging evidence which suggests the utility of artificial intelligence (AI) in the diagnostic assessment and pre-treatment evaluation of thyroid eye disease (TED). This scoping review aims to (1) identify the extent of the available evidence (2) provide an in-depth analysis of AI research methodology of the studies included in the review (3) Identify knowledge gaps pertaining to research in this area.

**Methods:**

This review was performed according to the 2020 Preferred Reporting Items for Systematic Reviews and Meta-Analyses statement (PRISMA). We quantify the diagnostic accuracy of AI models in the field of TED assessment and appraise the quality of these studies using the modified QUADAS-2 tool.

**Results:**

A total of 13 studies were included in this review. The most common AI models used in these studies are convolutional neural networks (CNN). The majority of the studies compared algorithm performance against healthcare professionals. The overall risk of bias and applicability using the modified Quality Assessment of Diagnostic Accuracy Studies 2 (QUADAS-2) tool led to most of the studies being classified as low risk, although higher deficiency was noted in the risk of bias in flow and timing.

**Conclusions:**

While the results of the review showed high diagnostic accuracy of the AI models in identifying features of TED relevant to disease assessment, deficiencies in study design causing study bias and compromising study applicability were noted. Moving forward, limitations and challenges inherent to machine learning should be addressed with improved standardized guidance around study design, reporting, and legislative framework.

## Introduction

Artificial intelligence (AI) is a term which refers to a branch in computer sciences that utilizes mathematical algorithms to attempt to perform tasks which usually require human cognition. In recent years, AI technology has advanced tremendously due to the enhancement of computational analytics techniques and the availability of large datasets. In healthcare, a substantial proportion of the AI literature is focused on medical imaging, where sophisticated algorithms are employed to develop models to improve diagnostic accuracy in medical image interpretation (1).

Thyroid eye disease (TED) is the main extrathyroidal manifestation of Graves’ disease (GD) which develops in about 25-50% of patients with GD (2). The disease is autoimmune in etiology and is characterized by inflammation and extensive remodeling of the soft tissues surrounding the eyes (3). The pathogenesis of the disease involves loss of self-tolerance to thyrotropin receptor (TSHR) and insulin-like growth factor-1 receptor (IGF-1R), leading to activation of sub-populations of orbital fibroblasts which triggers an autoimmune cascade, causing expansion of retro-orbital fat and enlargement of extraocular muscles (4). Disease manifestations include redness and swelling of the conjunctivae and lids, forward protrusion of the globes (proptosis), ocular pain, debilitating double vision, and even sight loss due to compressive optic neuropathy or breakdown of the cornea (5). Known as the “Rundle’s Curve”, TED begins with an active inflammatory phase which usually lasts for 18 months to 2 years before plateauing to a fibrotic inactive phase (6). The conventional goal of management is for early detection and treatment of active TED with immunosuppressive therapy. Late complications of TED such as compressive optic neuropathy or exposure keratopathy may not respond to immunosuppression alone and may require urgent surgical orbital decompression. Rehabilitative surgeries such as orbital decompression, strabismus, and eyelid surgeries are usually carried out in a staged fashion when the disease course becomes inactive. The recent discovery of the IGF-1 inhibitor shows improvement in proptosis, strabismus, and vision in active and even inactive TED patients (7, 8). These are potential harbingers and present a new paradigm for TED management in the future. Unfortunately, the high cost and potential risk of permanent hearing loss limit its widespread use in many countries. Despite advances in treatment, a large proportion of patients remain undiagnosed before debilitating symptoms such as diplopia and exposure keratopathy occur, often leading to impairment of the quality of life despite treatment. Early diagnosis and treatment of TED thus becomes an important area of research.

The first step in the diagnosis of TED is a dedicated ophthalmological examination, then orbital imaging may be employed in selected clinical situations. Orbital imaging in TED may be performed for several reasons: 1) Diagnosis of dysthyroid optic neuropathy (DON): This is a serious complication of TED which is sight threatening. Early recognition and treatment may avoid loss of sight 2) Diagnosis of TED with an atypical presentation: Although the diagnosis of TED is generally straightforward in a hyperthyroid patient, other differentials may need to be considered when the patient is euthyroid or hypothyroid or presents with individual signs such as isolated proptosis (e.g. due to lymphoma, cavernous sinus fistula), which may occur in 20% of all TED patients (9) 3) Evaluation of disease activity for prediction of therapeutic efficacy with anti-inflammatory and monitoring of treatment response.

There is emerging evidence which suggests the utility of AI in the diagnostic assessment and pre-treatment evaluation of TED. This scoping review aims to (1) identify the extent of the available evidence (2) provide an in-depth analysis of AI research methodology of the studies included in the review (3) Identify knowledge gaps pertaining to research in this area (10).

## Materials and methods

A literature search was performed by two independent investigators (CL and SW) from the earliest year of indexing until February 2023. Disagreements were resolved by consensus. This review was performed according to the 2020 Preferred Reporting Items for Systematic Reviews and Meta-Analyses statement (PRISMA) (11). A systematic literature search was performed in PubMed, Google Scholar, and Clinicaltrials.gov. The following terms were connected using Boolean operators “and”, “or” “and/or”, “thyroid eye disease”, “thyroid orbitopathy”, “thyroid-associated orbitopathy”, “graves’ orbitopathy”, “graves’ ophthalmopathy”, “machine learning”, “deep learning”, “artificial intelligence”, “convolutional neural network”. The terms were searched as “Mesh terms” and as “all fields” terms.

**Figure 1:**
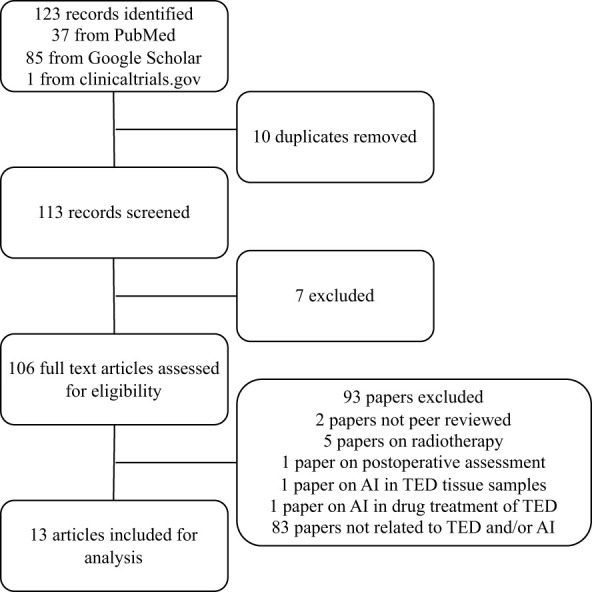
PRISMA flow diagram of included studies.

The search generated 123 abstracts, 10 of which are repeats, and the remaining 113 were individually assessed for suitability. Only full original articles of completed studies published in peer-reviewed journals that were written in English were included in this review. 13 artificial intelligence papers met the full inclusion criteria and were included (**Figure 1**). 7 papers were based on radiological scan images, 4 papers were based on external photographs of patients and 2 papers were based on clinical parameters.

### Tailored Quadas-2 tool for assessment of the quality of the AI studies

Given the absence of an internationally accepted AI-specific quality assessment tool for review papers, we adopted and tailored the QUADAS-2 assessment tool as recommended by the QUADAS-2 steering committee to improve its applicability in analyzing AI papers. QUADAS-2 determines the risk of bias and the applicability of each study in four main areas: patient selection, index test, reference standard, and flow and timing (12). These domains were assessed by using signaling questions with yes, no, and unclear answers. However, given the nature of the machine learning methods, we found the signaling question under “patient selection” on whether the study has avoided a case-control design to be redundant, thus this was assigned as non-applicable. Specific AI-related signaling questions were added (13, 14) to the domains of patient selection, index test(s), and reference standard (**Table 1**). Two reviewers independently judged the quality of each study. Disagreements were resolved by consensus with additional input from the third reviewer from the study team.

**Table 1:** Additional QUADAS-2 signalling questions tailored for this review of the AI literature.

DOMAIN 1: PATIENT SELECTION
A. Risk of Bias
Was the data in house or well curated open-source data?
Was the rationale and breakdown in the train, validate and test set described?
Is more than one institution included?
Did the study consider label imbalance if handling a classification problem?
Did the patient sample include an appropriate spectrum of patients to whom the diagnostic test will be applied in clinical practice?

DOMAIN 2: INDEX TEST(S)
A. Risk of Bias
Was the test evaluated against an external dataset?
Was overfitting avoided?
Was sufficient detail given on the algorithm to allow replication and independent validation?
Is there a specific design for end-user interpretability, e.g., saliency or probability maps?

DOMAIN 3: REFERENCE STANDARD
A. Risk of Bias
Was there a good expertise level and is there consensus amongst experts if used for performance benchmarking?

## Results

A total of 13 studies related to TED assessment for diagnosis and pre-treatment evaluation were included in this review. A summary the main clinical and patient demographic features of the studies is presented in **Table 2** and the full study characteristics are provided in **Table 3**. The number of patients recruited in the studies ranges from 108 to 2154 and main patient demographic details such as age, gender and smoking status were detailed in 8 of the 13 studies reviewed. The most common AI models used in these studies are convolutional neural networks (CNN). Three of the studies validated algorithms on external datasets (18, 23, 26). 12 of the 13 studies compared algorithm performance against healthcare professionals, whereas one study utilizes electronic medical records (EMR) phenotypes (15). Definitions of TED and threshold for diagnosis were generally based on current accepted clinical standards for all the studies included in this review. Less than half of the studies stated the method for internal validation and four studies described study design with end-user interpretability in mind (18, 19, 22, 23).

**Table 2:** Summary of the main clinical and patient demographic features of the studies.

No	Author	Main clinical and patient demographical features
**1**	Chaganti et al/2017 (15)	788 patients in disease cohort, of which 73 had TED vs 1566 controls who had cochlear implants
**2**	Salvi et al/2002 (a) (16)	Inactive vs Active TAO: 246 vs 152. No differences in age between the groups but women with active TAO were older
**3**	Salvi et al/2002 (b) (17)	Inactive vs Active TAO vs normal: 129 vs 113 vs 103 patients. Mean age (yrs): 47.1 ± 1.3 vs 49.5 ± 1.3 vs 48.4 ± 2.1 years. M:F 1:5 vs 1:6.5 vs 1.1. Immunosuppressive (Inactive vs Active TAO): 2.1% vs 7.1%. Smokers (Inactive vs Active TAO): 37.2% vs 43.4%.
**4**	Song et al/2021 (18)	TAO vs controls: 193 vs 715
**5**	Hu et al/2022 (19)	Training cohort: Steroid responsive vs Steroid unresponsive: 44 vs 34. Mean age (yrs): 47.6 ± 11.6 49.4 ± 10.6. M/F: 15/29 vs 18/16. Disease duration (months): 5.6 ± 4.4 vs 7.6 ± 5.3. Smokers’ vs Non-smokers: 14/30 vs 15/19. Euthyroid vs Non-euthyroid: 37/7 vs 28/6. CAS score: 3.6 ± 0.7 vs 3.7 ± 0.9 Validation cohort: Steroid responsive vs Steroid unresponsive: 18 vs 14. Mean age (yrs): 48.0 ± 13.5 vs 49.9 ± 11.4. M/F: 9/9 vs 6/8. Disease duration (months): 3.9 ± 2.5 vs 6.8 ± 3.4. Smokers vs Non-smokers: 5/13 vs 4/10. Euthyroid vs Non-euthyroid: 15/3 vs 12/2. CAS score: 4.2 ± 0.9 vs 3.7 ± 1.0
**6**	Lin et al/2021 (20)	108 patients with TAO. M/F: 42/66. Patients with active TAO were treated with immunosuppressives and MRI orbits of these patients were compared before and after treatment
**7**	Huang et al/2022 (21)	Active vs Quiescent vs Mild TAO: 487 vs 1073 vs 89. Moderate vs severe vs very severe TAO: 89 vs 1290 vs 181. M/F (total): 563/997. Majority of cases were below 50yo: 1055/1560
**8**	Hanai et al/2022 (22)	Enlarged extraocular muscle vs normal extraocular muscle: 199 vs 172. Mean age (yrs): 55.9 ± 13.7 vs 52.6 ± 18.4. M/F: 56/143 vs 40/132
**9**	Karlin et al/2022 (23)	TED vs controls: 829 vs 1459
**10**	Lee et al/2022 (24)	*Mild vs Moderate-to-severe GO vs controls: 99 vs 94 vs 95. Mean age (yrs): 38.4 ± 10.4 vs 47.6 ± 15.0 vs 29.3 ± 8.1. M/F: 13/86 vs 45/49 vs 37/58.
**11**	Wu et al/2022 (25)	TAO patients with DON vs TAO patients without TAO vs controls: 42 vs 49 vs 87
**12**	Shao et al/2022 (26)	TAO vs normal: 74 vs 74. Mean age (yrs): 43.76 ± 13.69 vs 43.28 ± 12.84. M/F: 17/57 vs 17/57. In the TAO group, 38 patients (51.35%) were diagnosed with bilateral TAO, and 36 patients were diagnosed (48.65%) with unilateral TAO. The TAO group consisted of 67 patients with hyperthyroidism, 4 patients with euthyroidism, 2 patients with Hashimoto thyroiditis, and 1 patient with primary hypothyroidism
**13**	Moon et al/2022 (27)	1020 patients with TAO. Mean age: 45.2 ± 15.4 years. M: F: 301/719. Mean total CAS score (available for 918/1020 patients): 2.0 ± 1.3. Active TAO (CAS ≥ 3) was observed in 272 patients (29.6%), and highly active TAO (CAS ≥ 5) in 34 (3.7%).

*Patient demographics were based on the 288 CT images used for this study. Significant differences in age and gender of the 3 patient groups.

GO, Graves’ orbitopathy; TAO, Thyroid associated orbitopathy; M/F, Number of males/ Number of females; DON, dysthyroid optic neuropathy.

**Table 3:** Is a large table, submitted as supplementary materials.

Study	Author/yr	Aim of study	No. patients	AI model	Image type	Reference standard	Internal validation	External validation	Outcome measures
**1**	Chaganti et al / 2017 (15)	Improve accuracy of AI model in classifying various optic nerve conditions with the addition of EMR phenotypes	2154	Boosted random forest	CT orbits CT performed for cochlear implants (controls)	NA	NA	No	AUC 0.81 With addition ofEMR phenotype: AUC 0.85
**2**	Salvi et al / 2002 (a) (16)	Classification and progression prediction of TED	398	3-layer neural network	Clinical ophthalmologic assessment and orbital CT or US	Expert	Hold-out	No	Classification: SN 86.2%, SP 80.2% Prediction of progression: SN 75.3% SP 52.2% Concordance between clinical assessment and neural network prediction: 67%
**3**	Salvi et al / 2002 (b) (17)	Classification and progression prediction of TED	345	3-layer neural network	Clinical ophthalmologic assessment and orbital CT or US	Expert	Hold-out	No	Correctly classified 78.3% of 115 eyes (87 patients) and predicted TAO progression in 69.2% of 39 eyes (28 patients)
**4**	Song et al / 2021 (18)	Screening of TED	908	Modified 3D-ResNet-18	CT orbit	Experts	Hold-out	Yes	AUC 0.919 Accuracy 87% SN 88% SP 85% Accuracy was 85.67% in the AI group and 84.33% in the resident group in the non- inferiority experiment
**5**	Hu et al / 2022 (19)	Value of T_2_WI-derived radiomics for pre-treatment determination of therapeutic response to glucocorticoids	110	Logistic regression (LR), decision tree (DT), support vector machine (SVM)	MRI orbits	Experts	Hold-out	No	LR achieved the best performance; Validation set results: AUC 0.916, Accuracy 87.5%, SN 86.1%, SP 89.3%, PPV 91.2%, and NPV 83.3% Integration of radiomics signature and disease duration: AUC 0.952, Accuracy 87.5%, SN 91.7%, SP 82.1%, PPV 86.8%, NPV 88.5%
**6**	Lin et al / 2021 (20)	Identification of active TED	108	CNNs built with blocks from VGG and ResNet	MRI orbit	Expert	Hold-out	No	Network (A): Accuracy 86.3%, SN 75.3%, SP 89.6%, Precision 68% Network (B): Accuracy 85.5%, SN 82.1%, SP 86.5%, Precision 64% Both network (A) and (B): AUC 0.922
**7**	Huang et al / 2022 (21)	Detect signs of TED based on facial images	1560	Single-shot multibox detector(SSD), U-net, and ResNet-50	Facial images	Expert	Hold-out	No	The mean AUC of the seven signs of TED :0.85, mean sensitivity 80%, mean specificity of 79%
**8**	Hanai et al / 2022 (22)	Detection of enlarged EOM	371	ResNet-50, VGG-16	CT orbit	Experts	Hold-out	No	AUC: 0.946 SN 92.5%, SP 88.6%
**9**	Karlin et al / 2022 (23)	Detect TED using external photographs	2288	An ensemble model of five ResNet-18	Facial image	Experts	Hold-out	Yes	Accuracy 89.2%, specificity: 86.9%, recall 93.4%, precision 79.7%
**10**	Lee et al / 2022 (24)	Diagnosis and severity assessment of TED	300	custom built CNN	CT orbit	Experts	Hold-out	No	Moderate-severe vs normal: AUC 0.979, Accuracy 0.930 Mild TED vs normal: AUC 0.895, Accuracy 0.826 Moderate-severe vs mild vs normal: AUC 0.905, Accuracy 0.842
**11**	Wu et al / 2022 (25)	Prediction of DON in TED	178	Double multiscale and multi attention fusion module + EfficientNetB0	CT orbit	Experts	Hold-out	No	Accuracy 96% SN: 94% SP: 99.5%, Precision: 98.9%
**12**	Shao et al / 2022 (26)	Automatic measurement of eyelid morphology in TAO patients	148 (separate 30000 images (celebA) to train eye detection model and 1862 healthy volunteer images to train eye segmentation model	R2AU-Net	Facial images	Experts	Hold-out	Yes	Accuracy: 98.5%
**13**	Moon et al / 2022 (27)	Assess CAS and diagnose active TAO	1020	linear kernel SVM (integrated with linear kernel PCA)	Facial images	Experts	Hold-out	No	SN: 72.7% SP 83.2% (entire dataset) SN: 88.1% SP 86.9% (dataset with consistent results for the 3 ophthalmologists SN: 40% SP: 49.9% (combination of above 2 datasets)

The full study characteristics of the 13 studies in this review. SN, sensitivity; SP, specificity; AUC; area under curve.

### Studies based on diagnostic imaging

There were seven studies that utilizes diagnostic imaging (CT or MRI orbits) in the diagnosis and severity assessment of TED (18, 24), identification of DON (15, 25), detection of disease activity (20) or enlarged extraocular muscles (22) and prediction of therapeutic response to glucocorticoid therapy (19). The area under the curve (AUC) of these AI systems ranges from 0.81 to 0.979, sensitivity (SN) from 75.3% to 94%, specificity (SP) from 85% to 99.5% and accuracy from 82.6% to 96%.

Song et al. reported an AUC of 0.919, with non-inferiority of the AI system demonstrated when compared to the resident group in diagnosing TED (18). The authors demonstrated higher sensitivity when they compared their 3D-Res Net (28) model to AlexNet and VGG thereby concluding its effectiveness in TED screening. The study utilizes class activation mapping (CAM) for transparency of the CNN. However, >70% of the database were patients with moderate to severe TED, and the judgment from residents rather than senior experts were used in this study. Lee et al. developed a new neural network for the diagnosis and severity assessment of TED, with a reported AUC of 0.979 for moderate to severe TED and 0.895 for mild TED (24). The performance of the new neural network was better than that of GoogLeNet, ResNet-50, Visual Geometry Group-16 (VGG-16), and even three oculoplastic surgeons, although details were not clear on the exact matrices that the experts based their decision-making on. In both studies, CT images were pre-processed via methods such as cropping, rotation or segmenting based on HU levels targeting extraocular muscles or fats.

Two studies developed AI models to identify DON (15, 25). Chaganti et al. showed improvements in the AUC of their AI model in classifying various optic nerve conditions, including DON in TED, when electronic medical records (EMR) information was incorporated into CT imaging data (15). Adding EMR phenotypes (derived from an EMR-based phenome-wide associated study (PheWAS) to imaging markers increased the AUC from 0.81 to 0.85. Wu et al. developed a deep learning hybrid model which is composed mainly of the double multiscale and multi-attention fusion module (DMs-MAFM) and a deep convolutional neural network for predicting suspected DON using CT orbits (25). The dataset was obtained from 178 patients, of which only 42 had DON. The model was trained on an augmented set of coronal views of the orbits at various distances from the interzygomatic line. The hybrid model reached a high accuracy rate of 96%, sensitivity of 94%, specificity of 99.5% and precision of 98.9%.

Lin et al. constructed DL systems based on CNN to distinguish active and inactive TED using 160 MRI orbit images (20). Network A inherited from the VGG network (29) and network B was constructed with the addition of parts of the Residual Neural Network (28). Both networks achieved high accuracy (network A 0.863 ± 0.055, network B: 0.855 ± 0.018). After modification, network B improved the sensitivity (0.750 ± 0.136 to 0.821 ± 0.021). The AUC of both networks was 0.922.

Hanai et al. developed a diagnostic software system to evaluate enlarged extraocular muscles (EEM) in TED patients using the orbital coronal CT data from 199 patients with EEM and 172 controls with normal extraocular muscles (22). The system was constructed based on a deep neural network using ResNet-50 (28) and VGG-16 (29). *Post-hoc* explainability was achieved using Score-CAM to construct heat maps for indicating where images in the convolutional neural network were focused. The system demonstrated a sensitivity of 92.5%, specificity of 88.6%, and AUC of 0.946.

Hu et al. performed radiomic analysis of MRI T2-weighted (T2w) coronal orbital images using the eight most identifiable features, all of which were related to signal intensity or heterogeneity (19). The study also found that higher minimal T2w signal intensity of the extraocular muscles, corroborated with earlier literature findings that they were more responsive to treatment, presumably due to higher water content. Integration of radiomics signature and disease duration further improved the diagnostic performance when compared to radiomics signature only (AUC of validation set improved from 0.916 to 0.952). Radiomics feature inputs with clinical value providing interpretability in these AI models. This study, however, lacked a test set to verify the model’s applicability to internal or external data and it only assesses active, moderate-to-severe TED.

### Studies based clinical assessments

Two studies [Salvi et al. (a) and (b)] by the same group utilized clinical assessments in combination with US or CT orbit in TED diagnosis and prediction of disease progression (16, 17) of which only one study provided the sensitivity and specificity of the AI system (16). Both studies recruited patients already known to have GD at the ophthalmology clinic in a single institution. It is unclear if there were appropriate exclusion criteria. Training set data was provided by an expert clinician (ophthalmologist and endocrinologist). However, there was no internal or external validation used for the studies. Concordant classification between AI and experts occurred in 86.2% and 78.3% and the ability to predict the progression of disease was 67% and 69.2% respectively, although the number of patients that progressed in both studies was small.

### Studies based on digital facial images

Four studies utilized facial photos to detect signs of TED (21, 23, 26) or assess disease activity (27). Outcomes assessed were heterogenous amongst studies: diagnosis of TED (23) presence of severe signs of TED (21), eyelid morphology in TED (26) and prediction of CAS score (27).

In the study by Karlin et al, compared to the expert clinician, the deep learning ensemble model demonstrated higher recall (89% vs 58%) but lower specificity (84% vs 90%) than the pooled expert cohort in detecting TED using facial images (23). The study utilized Grad-CAM to perform heatmap analysis of a component neural network model within the ensemble and found pixels corresponding to the eye and periocular region most strongly activate the TED class. Shao et al. developed a fully automatic computer-based assessment system to measure eyelid morphology in patients with TED (26). Manual measurement of margin to reflex distance (MRD) 1 and 2 by experienced ophthalmologists was compared to this automatic system. The intraclass correlation coefficients (ICCs) used to assess the agreement between automatic and manual measurement of MRDs demonstrated ICC of 0.980 for MRD1 and 0.964 for MRD2 in TED eyes, and ICC of 0.967 for MRD1 and 0.932 for MRD2 in control eyes, with ICC between repeated automatic measurements of MRDs up to 0.998, reflecting a strong agreement between the two, with high repeatability demonstrated in the automatic system. The diagnostic system for TED developed by Huang et al. accurately diagnosed TED via multiple task-specific models using facial images, with the ability to detect several signs of TED (21). The diagnostic methods used in this study included modules based on eye location (Module I), ocular dyskinesis (Module II), and other TED signs (Module III). Module I had an accuracy of 0.98; Module II had an accuracy of 0.93 for corneal segmentation and 0.87 for scleral segmentation. For Module III, the area under the receiver-operating curve (AUROC) for the detection of eyelid edema was 0.90, conjunctival congestion was 0.91 and eye movement disorders were 0.93. However, the diagnostic accuracy for TED signs that require auxiliary modalities to aid evaluation, such as chemosis and corneal ulcer, were lower (AUROC 0.60 and 0.70, respectively). The mean AUROC of the seven TED signs evaluated was 0.85, with a mean sensitivity of 0.80 and specificity of 0.79. Moon et al. developed an ML-assisted system for predicting CAS and diagnosing active TED using facial images (27). The system predicted CAS within 1 point of the reference CAS in 84.6% and 89% of cases when tested using the entire dataset and in the dataset with consistent results for the three ophthalmologists, respectively. However, the system showed differences in the performance of individual inflammatory signs, which could be further improved.

### Quality assessment

**Figure 2:**
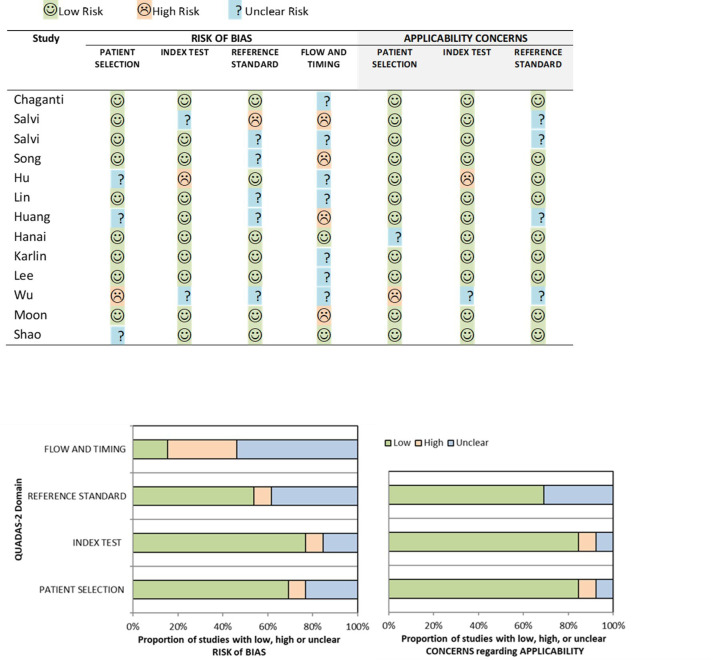
Risk of bias and applicability concerns summary about each modified QUADAS-2 domain presented as percentages for the 13 reviewed studies.

We performed a quality assessment of the 13 studies using a modified QUADAS-2 tool. The overall risk of bias and applicability using the modified QUADAS-2 tool led to most of the studies being classified as low risk, although higher deficiency was noted in the risk of bias in flow and timing (**Figure 2**). For patient selection, 9/13 (69%) studies had a low risk of bias. Most of these studies used in-house data following clinically established criteria for diagnosis, avoided inappropriate exclusions, and considered label balance in classification problems. However, for flow and timing a high or unclear risk of bias was seen in 11/13 (84.6%) of the studies. This was largely due to the unknown interval between the index test and the reference standard and whether all the patients received the same reference standard. For the reference standard domain, high or unclear risk was noted in 6/13 (46.2%) of the studies. This was mainly due to inconsistencies in the reference standard employed for the studies and concerns regarding the expertise level and level of consensus amongst experts when used for performance benchmarking. For the patient selection domain, high or unclear risk was noted in 4/13 (30.8%) of the studies. This was mainly due to a lack of description on the rationale for the breakdown of data into training, validation, and test set and whether pre-processing of data may significantly change the data set and reduce its applicability in testing on an external dataset. For the index test domain, high or unclear risk was noted in 3/11 (27.3%) of the studies, mainly due to a lack of description of the prespecified threshold settings.

## Discussion

Machine learning (ML) is a subdivision of AI that constructs data analytical algorithms to extract features from data. In medical applications, input data includes medical images and patient clinical data, which includes baseline data, disease-specific data, and disease outcomes. ML algorithms can be broadly divided into two major categories: unsupervised and supervised learning. Unsupervised learning is predominantly for feature extraction, while supervised learning is suitable for predictive modelling through building some relationships between the patient characteristics (as input) and the outcome of interest (as output) (30). In general, supervised learning provides more clinically relevant results; hence AI applications in healthcare, in medical imaging analysis, supervised learning is most often used. Traditional ML techniques such as linear regression, logistic regression (LR), random forest (RF), decision tree (DT), support vector machine (SVM), and neural network are feature-based supervised learning algorithms (31). For instance, Chaganti et al. employed RF classifiers comprising 100 trees to assess the diagnostic efficacy of image-derived features, phenotypes derived from electronic medical records, and clinical visual assessments (i.e., visual disability scores) in predicting optic nerve pathology (15). In another study by Hu et al, three machine learning models, namely LR, DT, and SVM were developed based on selected features to predict the response to glucocorticoid therapy in TED patients (19). Furthermore, Moon et al. proposed a submodel using linear kernel SVM integrated with linear kernel PCA. Subsequently, five such submodels, along with two consensus models (an aggregation model and a voting model), were designed for predicting the clinical activity score in TED (27). Traditional ML models offer interpretability with transparent decision-making, simplicity with well-defined theory, lower data requirements leading to computational efficiency, and robustness to noise due to explicit feature engineering. These characteristics contribute to the broad applicability of such models in the field of medical imaging analysis. However, several initial steps are necessary prior to the development of these AI algorithms, such as defining the image features to be extracted and selecting the region of interest (ROI), which needs to be done by field experts.

Deep learning (DL) is a subfield of ML and is an extension of the classical neural network technique whereby a cascade of multi-layered artificial neural networks for feature extraction and transformation. DL essentially imitates the neural connections made in the human brain. In recent years, DL has demonstrated exceptional performance across various domains, including computer vision and natural language processing (32). Leveraging the strong modelling capabilities of DL models, researchers have started to explore their application in TED-related tasks to achieve boosted performance. Some studies concentrate on utilizing Multilayer Perceptron (MLP) models. Notably, in the two studies by Salvi et al., a three-layer MLP architecture comprising an input layer, a hidden layer, and an output layer was employed to predict the progression of TED (16, 17). In both studies, the adoption of MLP models stems from their advantages over multivariate statistical analysis, as MLP models do not require explicit definitions of associations between features during modelling. Instead, they learned these associations in a data-driven learning process. Of various DL architectures, convolutional neural networks (CNN) are commonly applied for image recognition and computer vision applications because they preserve spatial relationships in 2D data, and thus outperform other architectures on image pattern recognition Researchers employ various CNN models to facilitate TED diagnosis and pre-treatment evaluation. For instance, Song et al. proposed the 3D-ResNet model (with the original 2D convolution modified to 3D), which incorporates residual connections to mitigate performance degradation caused by larger network depth (18). Lin et al. adopted two CNN models, one inheriting the VGG network (29) with smaller filters to reduce complexity, and the other utilizing ResNet (28) to address issues such as gradient vanishing and exploding (20). Huang et al. presented a system for TED diagnosis based on facial images, consisting of three modules: (i) a single-shot multibox detector for object detection (33), (ii) U-Net for semantic segmentation (34), and (iii) ResNet50 (28) for detecting TED signs (21). In the Hanai et al. study, the focus was on leveraging AI models to automatically detect enlarged extraocular muscles in TED using CT orbits. Their model combined Residual Network-50 (28) for segmentation and VGG-16 (29) for classification (22).

In addition to utilizing existing CNN models and techniques, researchers are actively exploring the development of novel model architectures tailored to specific application requirements. They aim to go beyond using off-the-shelf CNN models and techniques, seeking to address the unique demands of their research objectives. Karlin et al. introduced a novel approach for making use of external photographs to detect TED (23). They proposed an ensemble neural network model consisting of five neural networks, each employing a ResNet18 (28). The ensemble mechanism selected the output of the neural network among the five networks, which assigned the highest prediction probability for TED. Such an ensemble had a learning strategy that aims to achieve improved predictive performance while enhancing robustness to noise and outliers. By leveraging the collective decision-making power of multiple networks, the proposed ensemble model demonstrated potential advantages in thyroid eye disease detection. In the study conducted by Lee et al, the authors focused on improving the diagnosis and severity assessment of TED by modelling clinically routine orbital CT scans using neural networks (24). To address the challenge of incorporating CT images from axial, coronal, and sagittal views, which conventional CNN models cannot directly handle, they proposed a multi-view CNN model. This model was designed to process all three views simultaneously and comprised three sets of convolutional layers, a fully connected layer, and a classifier. By leveraging multi-view learning, the model could capture a comprehensive representation of the input data, leading to enhanced analytic performance in diagnosing and assessing the severity of TED. In the work by Wu et al, an efficient and convenient method was introduced for diagnosing DON (25). The authors proposed a hybrid model that combined the double multiscale and multi attention fusion module (DMs-MAFM) with EfficientNet B0 (35). The DMs-MAFM was built on the synergy between the multiscale feature fusion module (Ms-FFM), the multiscale channel attention aggregation module (MsCAAM), and the spatial attention module (SAM). This integration enhanced the model’s ability to attend to small objects and effectively extract features. By leveraging the DMs-MAFM and EfficientNet B0, the proposed hybrid model offered improved performance and convenience for diagnosing DON. Finally, in the study conducted by Shao et al, the authors focused on the image analysis of eyelid morphology in TED (26). They proposed a novel model called Attention R2U-Net (36), which combined a recurrent residual convolutional neural network with attention gate connections based on U-Net. The Attention R2U-Net aimed to achieve more accurate segmentation of eyelid morphology. The traditional convolutional block in the model was replaced with a recurrent convolutional unit to effectively capture low-layer features, resulting in improved performance and enhanced segmentation accuracy. By incorporating attention gate connections and leveraging the recurrent convolutional unit, the Attention R2U-Net model offered promising advancements in the analysis of eyelid morphology in TED. A summary of the various AI techniques used in the studies reviewed in this paper is presented in **Table 4**.

**Table 4:** Categorization of AI models used in TED studies.

AI Model Category	AI Model Subcategory	TED Studies
Traditional Machine Learning Models	Random Forests	Chaganti et al., 2017 (15)
	Support Vector Machine, Decision Tree, Logistic Regression	Hu et al., 2022 (19) Moon et al., 2022 (26)
Deep Learning Models	Multilayer Perceptron	Salvi et al., 2002 (a) (16) Salvi et al., 2002 (b) (17)
	Convolutional Neural Networks	Song et al., 2021 (18) Lin et al., 2021 (20) Hanai et al., 2022 (22) Huang et al., 2022 (21) Karlin et al., 2022 (23) Lee et al., 2022 (24) Wu et al., 2022 (25) Shao et al., 2022 (26)

This review aims to quantify the diagnostic accuracy of AI models in the field of TED assessment and appraise the quality of these studies using the modified QUADAS-2 tool. The QUADAS-2 tool is the most used instrument in the quality assessment of diagnostic accuracy studies (12) and its use is recommended by current PRISMA 2020 guidance (11). The tool provides transparency in the rating of the study bias and applicability in answering its review question. However, QUADAS-2 does not accommodate for specific terminology encountered in AI-related diagnostic test accuracy studies, nor does it educate researchers on the sources of bias found within this class of study (14). As such, we tailored QUADAS-2 components to better suit the quality assessment of studies related to TED diagnosis and pre-treatment evaluation based on the framework proposed by Soundarajah et al. (14) which addresses the unique potential biases related to AI-related diagnostic studies (**Table 1**).

To build a robust AI model, data quality is important. which requires an appropriately curated source. The data is more reliable if the data was collected in-house or from a well-curated open-source database since a poorly curated open-source database runs the risk of data duplication and erroneous labelling (14). Label imbalance should be addressed, particularly when identifying a rare disease (such as TED), to avoid the accuracy paradox problem that can result in a model with excellent accuracy but is inapplicable clinically (37). An appropriate spectrum of patients identified and the inclusion of more than one institution is favorable in building a model that is reproducible and beneficial to a wider community. For the assessment of the index test, we took into consideration if the test was evaluated with an external dataset that would assess its reliability performance; if solutions to avoid overfitting on the testing set were mentioned; if there were sufficient details provided about the algorithm to allow for independent validation, and specific design for end-user interpretability to see if the model is assessing the target rather than potential “noise”. In assessing the study’s reference standard, we evaluated if widely accepted clinical criteria for diagnosis were used, such as Bartley’s and Gorman (38) or EUGOGO (39) diagnostic criteria for TED. If benchmarking was performed against clinicians, we assessed if a suitable domain expert level was used. The AI models reviewed by this study have a relatively high diagnostic accuracy in identifying presence, activity and severity of TED, using either facial photographs or radiological images such as those derived from CT or MRI. The ML diagnostic systems can be used as a screening or diagnostic tool, potentially reducing barriers to accessing specialist care and contributing to earlier diagnosis and timely treatment. Clinically, radiological investigations are generally performed in patients with more severe TED, hence limiting its utility for TED screening. Radiological approach via CT or MRI may also be limited by cost, availability, and the exposure to ionizing radiation. However, based on the results of the studies included in this review, AI systems based on radiological investigations has potential use in early identification of dysthyroid optic neuropathy, active disease and disease progression, and predicting treatment response. Beyond screening and diagnosis, AI models utilizing orbital imaging may also aid in surgical planning, such as predicting appearance change with orbital decompression surgery (40).

On the other hand, digital facial images-based AI systems have potential use in TED screening and disease monitoring, which can be adapted to mobile devices and cloud services, providing automated and remote diagnostic services for patients with TED. It may also be used for screening of TED in patients with autoimmune thyroid diseases. These systems could serve as a telemedicine screening tool to identify TED in patients with diverse phenotypical characteristics, irrespective of their care location. This has important implications in the remote patient monitoring, enabling early intervention and enhancing patient care. However, a greater training set including photographs of patients with differentials of TED, such as lid retraction (e.g., previous eyelid surgery) or conjunctival chemosis (e.g., carotid-cavernous fistula) will be required to improve the specificity of such a model.

Although we remain optimistic that such AI technologies will eventually be adopted at a large scale to benefit TED patient care, ML is not without its challenges and controversies. One of the criticisms of DL models is the black box paradigm, in which the internal workings of how the output classification, in this case, TED, is determined by the model is unknown. This is the so-called black box phenomenon and could eventually lead to a reduced acceptance of this technology by clinicians (41). There are several strategies used to help people gain insight into how these models work, including the use of Class Activation Mapping (CAM) and saliency map. For example, Song et al. used CAM to highlight areas of the CT scan deemed important by its model to diagnose TED and it revealed this to be at the anterior aspect of the orbits. The prevalence of TED is much lower than other ocular diseases, such as diabetic retinopathy and cataracts. Thus, the next challenge arises from the limited small number of available training samples to build robust models without suffering from overfitting i.e., the predicting model learns exactly the training set but fails to fit new data from the test. For example, the ability to distinguish patients other than moderate-severe TED may be challenging in the proposed AI model by Song et al. due to the smaller dataset of mild TED cases (18). Similar challenges are faced in AI models developed to detect DON due to the scarcity of sample data (25). Various strategies have been described to try to mitigate this challenge (42). In the studies reviewed in this paper, techniques such as data augmentation and transfer learning, which is essentially the use of pre-trained networks (typically on natural images) to circumvent the (perceived) requirement of large datasets for deep network training (43). For example, in the study by Shao et al, they trained the eye detection model using 30,000 facial images with landmark locations of the eye extracted from the CelebFaces Attributes Dataset (26) and in the study by Karlin et al, CNN was pre-trained on ImageNet, a large, labelled collection of low-resolution color photographs (23). While such strategies reduce the chance of overfitting the model, its applicability here to an external test set and clinical setting has yet to be tested. Ethnic differences exist in TED phenotypes, related to orbital and lid anatomy, genetic background and autoimmune responses (44). Hence, AI models trained based on Caucasian data may not be applicable to Asians, and vice versa. Datasets employed in the studies in this review were relatively small and detailed patient demographics and clinical findings were not available for several studies (**Table 2**), potentially limiting the generalizability and reproducibility of the developed ML algorithms to other patient populations. This underscores the significance of training and assessing models using extensive and varied datasets through collaborations and data pooling from multiple institutions and publicly available datasets to enhance its performance. The majority of the studies in this review evaluate AI systems in either imaging or facial images alone. The integration of clinical assessments, serologic markers and imaging features could be used to further enhance the reliability of these AI models and should be explored in future studies. Other limitations inherent to AI systems using digital facial images highlighted by several authors’ studies include the influence of imaging environments on diagnostic accuracy, the need for larger amounts of data to further improve the performance of the AI platform and inherent limitations of using 2-dimensional photos. Lastly, ethical and legal implications need to be considered when implementing AI solutions in clinical practice. Ethical challenges include (1) informed consent to use data, (2) safety and transparency, (3) algorithmic fairness and biases, and (4) data privacy. On the other hand, legal challenges include (1) safety and effectiveness, (2) liability, (3) data protection, and privacy, (4) cybersecurity, and (5) intellectual property law (45). It is therefore crucial that the relevant stakeholders and regulatory authorities collaborate to overcome these challenges to ensure successful implementation of any proposed AI solutions to benefit a wider community.

## Conclusion

The application of AI in clinical practice has enormous promise to improve the care of patients with TED. This review appraised the quality of the literature and quantified the diagnostic accuracy of AI models in the field of TED assessment. While the results of the review showed high diagnostic accuracy of the AI models in identifying features of TED relevant to disease assessment, several knowledge gaps in the current research in this area were identified through this review when the studies were objectively critiqued with our modified QUADAS-2 tool. Deficiencies in study design causing study bias such as small datasets, label imbalance and lack of external validation of the AI models, compromising study applicability were noted. Moving forward, these limitations and challenges inherent to ML should be addressed with improved standardized guidance around study design, reporting, and legislative framework for successful implementation in clinical practice.

## Data availability statement

The original contributions presented in the study are included in the article/supplementary material. Further inquiries can be directed to the corresponding author.

## Author contributions

C-LC: Conceptualization, Data curation, Formal Analysis, Investigation, Methodology, Project administration, Resources, Writing – original draft, Writing – review & editing. KZ: Formal Analysis, Investigation, Methodology, Writing – original draft, Writing – review & editing. AK: Investigation, Writing – original draft. M-HL: Investigation, Writing – original draft. DT: Supervision, Writing – review & editing. CW: Investigation, Writing – original draft. GH: Formal Analysis, Methodology, Writing – original draft. BO: Supervision, Writing – review & editing. SK: Conceptualization, Data curation, Formal Analysis, Investigation, Methodology, Writing – original draft, Writing – review & editing.
